# Peripheral Seal Composite Barrier (PSCB) for Management of Palatal Donor Sites in Mucogingival Surgery: A Randomized Clinical Trial

**DOI:** 10.1002/cre2.70470

**Published:** 2026-10-06

**Authors:** Moein Khojaste, Farid Shiezadeh, Zahra Moslehitabar

**Affiliations:** ^1^ Department of Periodontics, School of Dentistry Mashhad University of Medical Sciences Mashhad Iran; ^2^ Dental Research Center Mashhad University of Medical Sciences Mashhad Iran

**Keywords:** autograft, composite resins, hemostasis, pain management

## Abstract

**Objectives:**

Management of the palatal donor site following periodontal plastic surgery remains challenging because of postoperative pain, bleeding, and patient discomfort. This study aimed to evaluate the efficacy of a chairside‐fabricated composite barrier in reducing morbidity at the palatal donor site.

**Materials and Methods:**

In this randomized, parallel‐group clinical trial, 50 patients undergoing soft tissue augmentation were allocated to either a test group (composite barrier + collagen sponge) or a control group (collagen sponge alone). Intraoperative measurements included palatal tissue thickness, graft dimensions, and donor site management time. Postoperative outcomes (pain, bleeding, discomfort, chewing difficulty, stress, analgesic consumption, and willingness for retreatment) were assessed at prespecified time points during the first 2 postoperative weeks. Donor sites complications were specifically assessed on postoperative day 7.

**Results:**

All participants completed the study without dropouts. Compared with controls, the composite barrier group required significantly shorter wound management time (210.42 ± 52.04 vs. 445.76 ± 77.43 s; *p* < 0.001) and lower analgesic consumption during the first week (1328.00 ± 846.33 vs. 4160.00 ± 1366.26 mg; *p* < 0.001). Pain scores were significantly lower in the composite barrier group on postoperative days 1–7 (*p* < 0.001), slightly higher on day 10 (*p* = 0.007), and similar on day 14. Bleeding scores were lower during the first 4 postoperative days (*p* < 0.001). Patient‐reported discomfort, chewing difficulty, and stress were significantly reduced in the composite barrier group (*p* < 0.001), which also showed greater willingness for retreatment (7.80 ± 1.15 vs. 2.04 ± 1.34; *p* < 0.001). No necrosis, infection, or hematoma occurred.

**Conclusions:**

Application of a composite barrier for palatal donor site management reduces the time required to achieve intraoperative hemostasis and is associated with lower postoperative pain, bleeding, and patient discomfort. This approach may improve patient‐reported outcomes following periodontal plastic surgery.

**Trial Registration:**

This project was registered in the Iranian Registry of Clinical Trials under the ID (IRCT20250513065721N2), and the registration date was 21/6/2025.

**Abbreviations**
ASTG: autogenous soft tissue graft
CONSORT: Consolidated Standards of Reporting Trials
CTG: connective tissue graft
DGG: de‐epithelialized gingival graft
FGG: free gingival graft
FMBS: full‐mouth bleeding score
FMPS: full‐mouth plaque score
PRF: platelet‐rich fibrin
PROMs: patient‐reported outcome measures
PSCB: peripheral seal composite barrier
SCTG: subepithelial connective tissue graft
VAS: visual analog scale

## 1 Introduction

Autografts remain the gold standard for treating teeth and implants mucogingival defects (Lu et al. 2020). Despite the availability of various soft tissue graft substitutes, including xenogeneic and allogeneic materials, autogenous soft tissue grafts (ASTGs), including de‐epithelialized/free gingival grafts (D/FGGs) and subepithelial connective tissue grafts (SCTGs), continue to be widely used in periodontal plastic surgery (Thoma et al. 2014). Connective tissue grafts (CTGs) are often favored over free gingival grafts (FGGs) for phenotype modification and root coverage procedures, primarily because of better esthetic and color adaptation (Raoofi et al. 2019). The hard palate is commonly selected for soft tissue graft harvesting because of the superior characteristics of its keratinized gingiva (Parvini et al. 2021).

Harvesting a FGG from the palate results in an open wound that heals by secondary intention, often leading to significant postsurgical pain, morbidity, and challenges in intraoperative and postoperative bleeding control (Farnoush 1978). To achieve primary intention healing when a subepithelial CTG is harvested, various techniques have been proposed, including the trap door technique (Edel 1974), single incision (Hürzeler and Weng 1999; Lorenzana and Allen 2000), and parallel incision (Windisch and Molnar 2019) methods. Compared with FGG harvesting, these techniques are regarded as more conservative and less traumatic, reducing patient morbidity and discomfort (Wessel and Tatakis 2008). One drawback associated with these methods is lower CTG quality, especially in patients with thin palatal soft tissue; the densely structured connective tissue (lamina propria), which possesses superior properties, remains in the superficial palatal layer rather than being included in the graft (Zucchelli et al. 2010; Harris 2003). Furthermore, studies suggest that the residual soft tissue thickness covering the palatal bone plays a more significant role in postoperative pain than does the mode of healing (primary or secondary intention) itself (Zucchelli et al. 2010). In subepithelial connective tissue harvesting techniques, a considerable depth of palatal tissue is involved, and the surgical time is also increased, both of which may contribute to increased postoperative pain (Zucchelli et al. 2010; Griffin et al. 2006). In addition, it is not always feasible to use techniques that preserve the superficial epithelium and connective tissue as protective measures, especially when thin palatal mucosa (less than 3 mm thick) is present (Silva et al. 2023; Tavelli et al. 2023). Given these factors, the de‐epithelialized gingival graft (DGG) technique is now widely used. This method allows for the harvesting of higher‐quality CTG, which is mainly composed of the lamina propria and contains less fatty and glandular tissue, and better clinical outcomes (Tavelli, Ravidà, Lin et al. 2019). This technique also minimizes surgical time and preserves more palatal soft tissue thickness. Moreover, previous studies have reported a similar level of postoperative pain for the DGG approach compared with the subepithelial CTG technique (Zucchelli et al. 2010; Tavelli et al. 2023). However, the challenges associated with secondary intention healing, such as post‐operative pain, morbidity, and difficulty in bleeding control, remain significant.

Evidence suggests that patient's experience with ASTG affects their willingness to undergo future treatment (Tavelli et al. 2021). Thus, numerous studies have investigated the effectiveness of different methods for managing palatal wounds (Jankowski et al. 2024; de Almeida et al. 2024). These approaches aim to prevent excessive intraoperative and postoperative bleeding, accelerate healing, and reduce patient discomfort and pain (Silva et al. 2023). Examples include chitosan‐based dressings (Yadav et al. 2024), cellulose (Patarapongsanti et al. 2019), absorbable gelatin sponges (Rossmann and Rees 1999), platelet‐rich fibrin (PRF) (Mutallibli and Sağlam 2024), cyanoacrylate (Escobar et al. 2021), acrylic stents (Yussif et al. 2021), and propylene mesh (Yussif et al. 2021). However, there is still no gold standard approach for palatal wound management. Collagen sponges are among the most commonly used materials for wound management because of their tissue compatibility, nontoxicity, and cost‐effectiveness (Chattopadhyay and Raines 2014). They offer several benefits, including serving as a matrix for clot formation and organization (Guralnick and Berg 1948), accelerating hemostasis (Rossmann and Rees 1999), and promoting early healing of donor areas (Schinini et al. 2021). Recent studies on postoperative pain following graft harvesting have investigated the use of collagen sponges in combination with other products to enhance their effects (Meza‐Mauricio et al. 2023; Tavelli, Ravidà, Saleh et al. 2019). Compared with the use of collagen sponges alone, these approaches have been shown to more effectively reduce postoperative pain, discomfort, and analgesic consumption (Tavelli et al. 2018).

Given that clot stability is fundamental for successful wound healing, mechanical protection methods such as the custom‐made Hawley retainer and the modified Essix are considered the most effective approaches for wound protection and bleeding control (Eltas et al. 2014). Palatal stents and retainers can effectively reduce pain, discomfort, and bleeding while allowing for normal daily activities and social interactions (Yussif et al. 2021; Basma et al. 2023; Chiu et al. 2020). However, their effectiveness can be compromised by friction with the palatal mucosa during function, which may lead to stent dislodgement, delayed bleeding, pain, impaired healing, and speech difficulties (Yussif et al. 2021; Wyrebek et al. 2018). Additionally, the acrylic components of these appliances may cause tissue irritation and inflammation (Yussif et al. 2021), and their laboratory fabrication process is often time‐consuming and costly (Gisotti and Valente 2024). These limitations underscore the need for a simple, chairside‐fabricated, biocompatible alternative that can provide effective wound protection without compromising patient comfort or healing.

An alternative approach is the use of chairside‐fabricated composite barriers made from flowable composite resin (Meza‐Mauricio et al. 2023; Gisotti and Valente 2024; Laguna‐Martos et al. 2025). Previous studies have used composite shields for palatal wound protection. However, these shields were fabricated after graft harvesting and required prior hemostasis through pressure application and gelatin sponge placement, which made the process time‐consuming. Moreover, the composite barriers in previous studies were confined to the wound site and bonded to adjacent teeth, which required careful isolation and made the procedure more complex. In contrast, this study presents a practical refinement of existing methods, utilizing a preoperatively fabricated composite barrier, termed the peripheral seal composite barrier (PSCB), molded directly onto the palatal mucosa prior to graft harvesting. The PSCB extends beyond the wound margins and conforms to the surrounding tissue topography, enhancing stability, optimizing clot retention, and minimizing the risk of barrier displacement during healing. Moreover, the use of PSCB in conjunction with a collagen sponge may further enhance hemostasis and patient comfort. The primary aim of this randomized clinical trial was to evaluate the effectiveness of simultaneous PSCB and collagen sponge application in reducing postoperative pain and discomfort following free gingival graft harvesting.

## 2 Materials and Methods

### 2.1 Selection and Preparation of Participants

The present study was a prospective, parallel group, randomized, single‐center controlled clinical trial conducted at the periodontics department, School of Dentistry, Mashhad University of Medical Science, between June and July 2025. Figure 1 presents the Consolidated Standards of Reporting Trials (CONSORT) flow diagram, illustrating the patient enrollment process, allocation, follow‐up, and analysis. Fifty patients were randomly allocated into two groups: the test group, which received palatal protection with PSCB plus an absorbable collagen sponge, and the control group, in which only an absorbable collagen sponge was applied to the donor sites. This study lacked a third group with no treatment provided to the donor site for ethical reasons. Skipping the treatment for the donor site may lead to excessive pain and discomfort for patients, disregarding ethical research standards. The present study hypothesized that the PSCB technique would improve patient‐reported outcomes related to postoperative pain and discomfort. All eligible patients who participated in this study provided written informed consent. The patients were informed in detail about the risks, benefits, alternative treatments, steps involved, study timeline, and potential side effects of the treatment protocol. The study was conducted in accordance with the principles of the Declaration of Helsinki on human research ethics, as revised in 2013. This study was approved by the Ethics Committee of Mashhad University of Medical Sciences, Mashhad, Iran (Ethical code: IR.MUMS.DENTISTRY.REC.1404.032). This project was registered in the Iranian Registry of Clinical Trials under the identification number IRCT20250513065721N2 on 21/6/2025. This manuscript was prepared in accordance with the CONSORT 2025 reporting guideline (Hopewell et al. 2025). No significant modifications were made to the methodology, including eligibility criteria, treatment protocols, or outcome measures, after the trial began.

**Figure 1:**
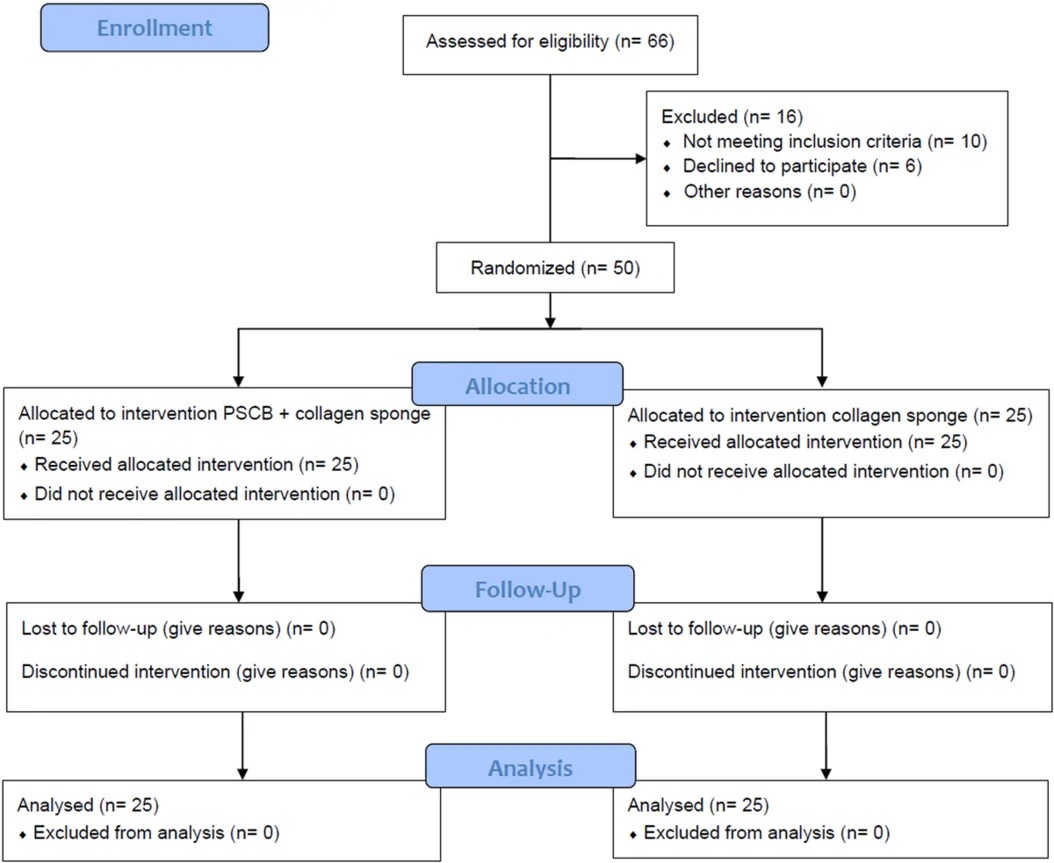
CONSORT flow diagram of patient selection and allocation.

Eligible patients were recruited from a pool of patients seeking mucogingival treatment. Participants were selected according to the inclusion and exclusion criteria mentioned below.

The following inclusion criteria were applied:

1. Age ≥ 18 years
2. Patients with gingival recessions require periodontal plastic surgery.
3. For the donor site, the minimum acceptable thickness of the palatal tissues was 2 mm (as measured via an anesthesia needle with an adjustable silicone stop and digital caliper), and the minimum acceptable height of the palatal vault was 12 mm (Reiser et al. 1996).

The exclusion criteria were as follows:

1. Current and former tobacco smokers;
2. Alcohol abuse‐related issues
3. Signs of active gingivitis or periodontitis
4. Full‐mouth plaque score (FMPS) and full‐mouth bleeding score (FMBS) ≥ 20% (probing of six sites per tooth)
5. Pregnant or lactating mothers
6. Patients with systemic diseases that contraindicated surgical treatment or affected tissue healing, psychiatric disorders, allergies, coagulation disorders (history of hemophilia, von Willebrand disease, or current anticoagulant therapy), and long‐term steroid therapy
7. Patients with a gagging reflex
8. Presence of oral lesions in the palate
9. History of previous palatal soft tissue harvesting
10. Ongoing orthodontic treatment with a palatal appliance
11. Patients with removable dentures covering the palate (maxillary arch)
12. Failure to complete the informed consent form

### 2.2 Sample Size Calculation

The sample size calculation was based on a previous study (Meza‐Mauricio et al. 2023) in which postoperative patient pain perception was considered the primary outcome. A power calculation was conducted with *α* = 0.05 and *β* = 0.20, corresponding to a power of 80%. On the basis of these parameters, a minimum of 20 patients per group were needed to achieve statistical significance. Owing to the possibility of dropout during the follow‐up period, 25 patients were assigned to each intervention group. Hence, 50 patients were included in this study.

### 2.3 Randomization, Allocation Concealment, and Blinding

Patients were randomly allocated to treatment groups via a block randomization approach with a block size of four to maintain balanced group distribution throughout the study. The randomization list was computer‐generated in advance by an independent researcher (Z.M.), who was not involved in the clinical procedures or data analysis. The same researcher prepared the sequentially numbered, sealed, coded, opaque envelopes containing the treatment allocations. To ensure that allocation remained concealed until the time of treatment, the operator (F.S.) opened the envelopes sequentially at the time of the intervention. This method reduced the risk of selection bias while maintaining the integrity of the randomization process.

This study followed a single‐blinded design. Blinding the patients was not possible because of the visible process of performing PSCB, and the surgeon performing the procedures was necessarily aware of the group assignments because of the differing management protocols for the donor site in the test and control groups. However, measures were taken to minimize potential bias through partial blinding: the examiner (M.K.) responsible for patient interviews and data collection during follow‐up sessions was blinded to group allocation and did not participate in the surgical procedures. Furthermore, the statistician analyzing the data received anonymized datasets without group identifiers, ensuring that the statistical analysis was conducted in a blinded manner.

### 2.4 Treatment Protocol

Presurgical phase: Preoperatively, basic clinical photographs, medical and dental histories, and radiographic examinations were performed to assess patient suitability for the study; 2 weeks prior to scheduled surgery, each patient received prophylaxis and periodontal therapy, consisting of full‐mouth supragingival scaling via ultrasonic and hand instruments as well as personalized oral hygiene instructions.

Surgical procedure: All patients had identical surgical methods (root coverage via the tunneling approach + CTG) performed by a single surgeon to reduce variations in the procedure. Also, all surgeries were performed under local anesthesia (2% lidocaine with 1:100,000 epinephrine) using the infiltration technique. Before surgery, patients were instructed to rinse with a 0.12% chlorhexidine mouthwash for 1 min. After the recipient site was prepared, a sterile aluminum foil template was created to match the desired graft dimensions. The template was placed on one side of the palate (opposite to the recipient site) distal to the palatal rugae area and 2 mm apical to the gingival margins to determine the incision outlines for harvesting the graft. For patients in the test group, after determining the appropriate size and location of the donor site and prior to graft harvesting, a chairside PSCB was fabricated. A micro‐hybrid flowable composite resin (DenFil, Korea) was used without prior etching or bonding procedures. As illustrated in Figure 2a–d, the composite resin was applied directly onto the palatal mucosa, extending approximately 2–3 mm beyond the planned graft margins to ensure complete peripheral coverage. Initially, a circumferential frame was created to delineate the intended harvesting area, followed by placement of two diagonal composite lines to subdivide the area into four equal segments. Each segment was then incrementally filled with flowable composite resin and light‐cured using an LED curing unit (Curing Pen, Eighteeth, China; output intensity 1500 mW/cm^2^) for 5 s per segment. After completion of all segments, an additional 10‐s curing cycle was applied over the entire PSCB to ensure complete polymerization and mechanical stability. This stepwise application resulted in a uniform composite barrier with an approximate thickness of 1–2 mm, providing adequate protection of the palatal donor site while maintaining patient comfort.

**Figure 2:**
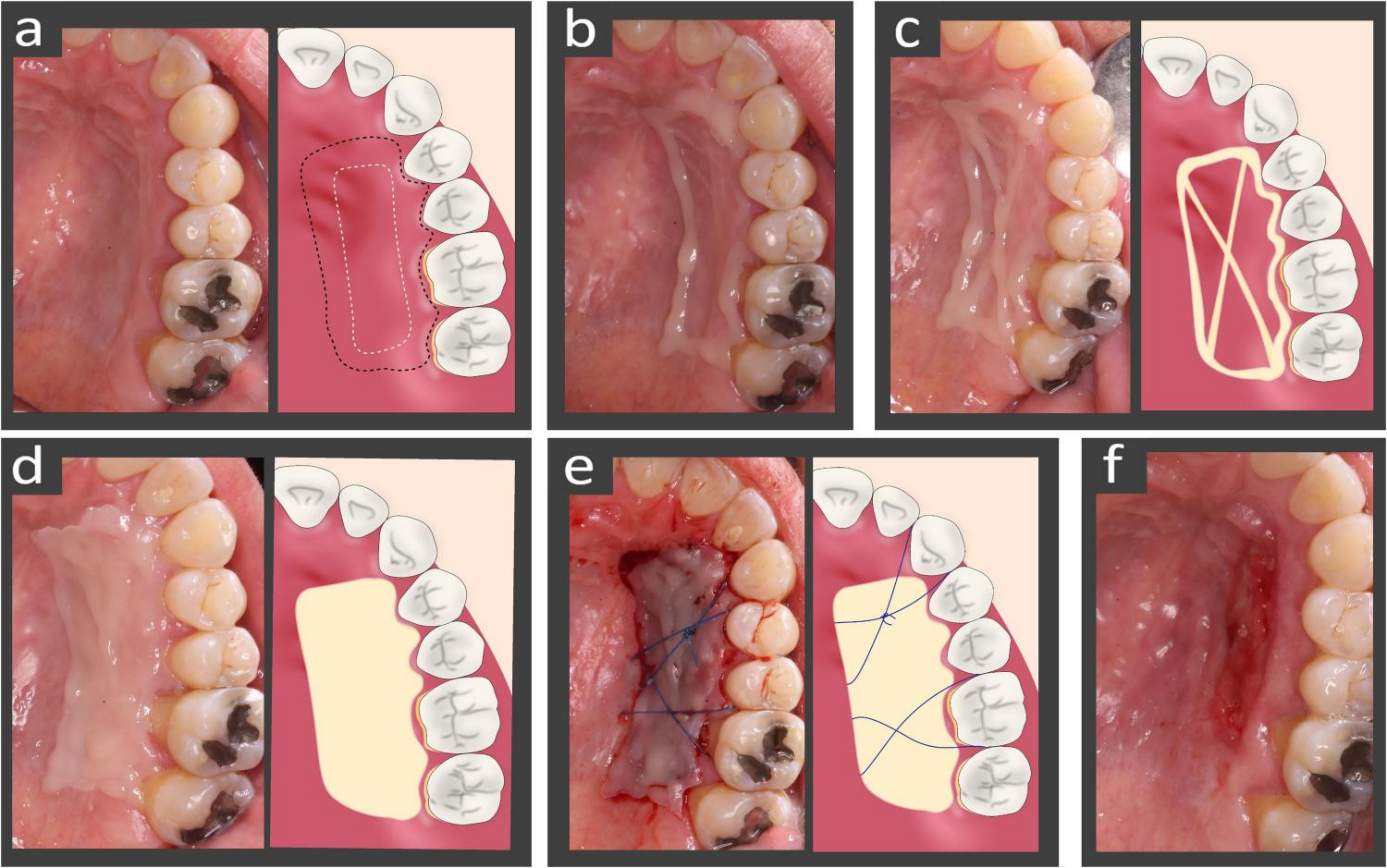
Procedures performed in the test group (clinical view and schematic illustration): (a) Preoperative view. (b) Formation of a circumferential composite frame around the predefined graft harvesting area on the palatal mucosa, extending approximately 2–3 mm beyond the intended graft margins to delineate the rectangular donor site. The composite frame was then light‐cured using an LED curing unit (Curing Pen, Eighteeth, China; 1500 mW/cm^2^) to stabilize the borders. (c) Placement of two diagonal composite lines subdividing the internal area into four segments, followed by light curing. (d) Incremental filling of each segment with hybrid flowable composite resin (DenFil, Korea), followed by light curing (5 s per segment), with an additional 10‐s curing cycle applied over the entire structure to ensure complete polymerization. This results in a uniform protective composite layer (~ 1–2 mm thickness). (e) Stabilization of the PSCB over a collagen sponge using sling cross‐mattress sutures around adjacent teeth. (f) Clinical view of the donor site at 7 days postoperatively, at the time of PSCB removal.

After determining the graft site, palatal mucosa thickness was measured at three points (along the long axis of the previously demarcated area at the mesial, central, and distal parts) via an anesthesia needle with a silicon stop and a digital caliper. The FGG was harvested following the surgical technique described by Zucchelli et al. (2010). Immediately after graft harvesting, the palatal wounds in both groups were covered with an absorbable hemostatic sponge (Spongostan, Ferrosan Medical Devices, Denmark). In the control group, the hemostatic sponge was kept in place by a sling cross mattress suture performed with 5–0 nonabsorbable monofilament thread (polyamide, Supa, Iran), which engaged the soft tissue apical to the palatal wound and anchored around the adjacent teeth (Figure 3a–c). In the test group, after placement of hemostatic sponge over the wound, the custom‐made PSCB was positioned and adapted to the site. It was then secured via a sling cross‐mattress suture with 5–0 nonabsorbable monofilament thread (polyamide, Supa, Iran) (Figure 2e). The sling sutures were placed in both groups by engaging the palatal tissue apical to the wound area. The donor site working time (i.e., surgical chair time for palatal wound management) was measured via a stopwatch, starting immediately after graft harvesting and ending with the placement of the final suture that achieved hemostasis and stabilization of the donor site. In the control group, this included the time required for applying the gelatin sponge, stabilizing it with sutures, and applying gauze pressure to control bleeding. In the test group, it included the time needed for applying the gelatin sponge and fixing the PSCB. Any additional time required to manage delayed bleeding at the end of the surgery, such as further pressure application or placement of extra sutures, was also added to the total working time. All patients were monitored for 30 min post‐operatively to ensure acceptable hemostasis and bleeding control before discharge. For all of the harvested grafts, a digital caliper and an endodontic spreader equipped with a rubber stop were used to measure the width (apicocoronal dimension), length (mesiodistal dimension), and thickness (in the mesial, central, and distal parts) of the graft before the removal of any fatty and glandular tissues.

**Figure 3:**
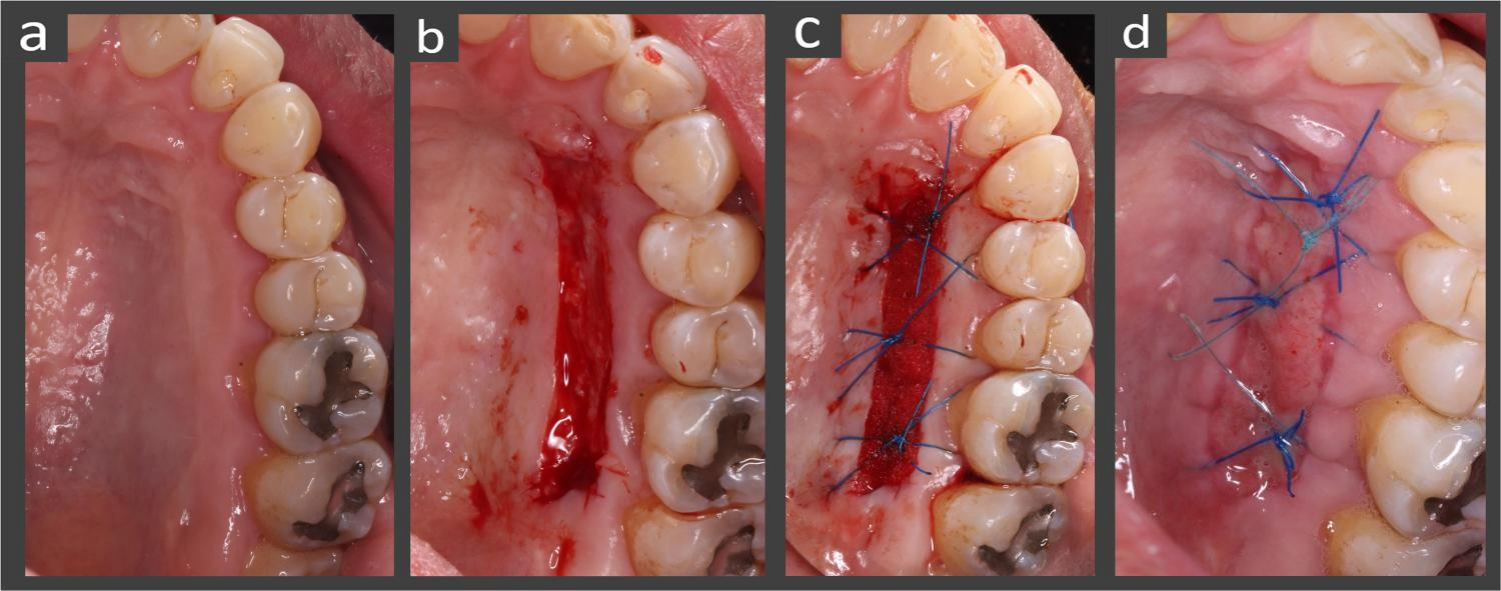
Procedures performed in the control group (clinical view): (a) Preoperative view. (b) Palatal wound after free gingival graft harvesting. (c) Protection of the palatal wound via a hemostatic collagen sponge stabilized by sling cross mattress sutures around the teeth. (d) Clinical view of the donor site 7 days post‐surgery.

### 2.5 Postoperative Care

Patients were examined at 1 and 2 weeks postoperatively, with donor‐site complications specifically assessed and documented at the 1‐week visit. Patients were instructed to avoid brushing the palatal aspect of the maxillary teeth until removal of the protective composite barrier and sutures. The PSCB and sutures were removed at 7 days postoperatively in all cases (Figure 2f and Figure 3d), with no instances of early removal. Pain was managed with 400 mg tablets of ibuprofen. Each patient received a sealed full pack of ibuprofen (Dana, Iran) tablets and was asked to return the pack to the follow‐up visit, where the number of tablets consumed was recorded to assess pain levels indirectly through analgesic consumption. Patients were instructed to take one tablet immediately after the procedure and one more tablet after 8 h. After that, they were advised to take additional tablets only if needed. Additionally, the participants were instructed to use a 0.12% chlorhexidine mouth rinse twice daily for 1 min for 2 weeks, starting from the day after surgery. Additionally, patients were advised to follow a soft and cold food diet for 4 weeks. Systemic antibiotics (500 mg amoxicillin three times a day for a week) were prescribed. Patients received both written and verbal postsurgical instructions. Sutures at the recipient site were removed after 2 weeks, and at that time, the use of an ultrasoft toothbrush and floss was recommended.

### 2.6 Study Variables

The primary outcome of this randomized, single‐blind controlled clinical trial was to evaluate and compare the effectiveness of PSCB in reducing patients’ postoperative pain, as well as its potential to decrease donor site working time, compared to a hemostatic agent alone. The secondary outcomes include evaluation of postoperative discomfort, chewing ability, stress, delayed bleeding, occurrence of complications (necrosis, infection, hematoma), and willingness for the same possible treatment in the future.

### 2.7 Clinical Assessments

- The donor site working time was defined as the duration of the surgical procedure required to achieve complete hemostasis and bleeding control of the donor site. It was measured using a stopwatch, starting immediately after graft harvesting and ending with the placement of the final suture that ensured bleeding control. All intraoperative measures required to achieve hemostasis—including initial placement of hemostatic materials, suturing, repeated gauze pressure application, and any additional interventions—were included within this time measurement. Intraoperative bleeding was not recorded as a separate variable; however, its presence and severity were indirectly reflected in the duration of the working time.
- Postoperative pain was evaluated by recording the total ibuprofen consumption (mg) during the first postoperative week. Additionally, a blinded examiner (M.K.) contacted each patient daily by phone during the first week and on postoperative days 10 and 14 to record pain intensity via the visual analog scale (VAS), which ranged from 0 (no pain) to 10 (worst pain experienced) (Price et al. 1983). Patients were instructed to report only pain related to the palatal donor site, without considering any discomfort at the recipient site.
- Delayed bleeding was defined as prolonged hemorrhage occurring within the first postoperative week. A blinded examiner (M.K.) contacted each patient daily during the first week and on postoperative days 10 and 14 to record the occurrence and severity of bleeding using VAS, where 0 indicated no bleeding, and 4 indicated continuous heavy bleeding (Lambade and Thorat 2017).
- Postoperative discomfort was defined by the degree of tenderness reported by patients in the first week following surgery, resulting from the palatal donor site, and its impact on speaking ability, work capacity, and quality of life. This score was measured using VAS at the first follow‐up visit at 7 days post‐surgery, with patients reporting their overall discomfort throughout the first postoperative week.
- Inability to chew was defined as the degree of change in the patient's eating habits due to the presence of a palatal wound. The VAS score was recorded 7 days post‐surgery, with patients reporting their overall change in dietary habits throughout the first postoperative week (VAS score ranged from 0: no change; 10: extreme change in chewing ability and dietary habits).
- Postoperative stress was defined as the level of anxiety and fear experienced by patients regarding the risk of jeopardizing the palatal wound. The VAS score was used to assess the patient's reported stress throughout the initial postoperative week on day 7 after surgery.
- The examiner (M.K.) conducted a clinical assessment of postoperative complications, such as necrosis, infection, and hematoma, 7 days post‐surgery and documented their presence or absence.
- Patients were requested to rate their readiness to undergo another palatal graft harvesting procedure on a 0–10 scale, where 0 signified “absolutely unwilling” and 10 indicated “indifferent” to repeat the surgery if an option existed (Basma et al. 2023). This evaluation was performed 1 week post‐surgery.

Graft length, width, thickness, and palatal soft tissue thickness were considered as cofactors.

- The graft length and width were assessed via an endodontic spreader equipped with a rubber stop. The distance from the spreader tip to the rubber stop was measured with a digital caliper (0.01 mm accuracy).
- The thickness of the graft was assessed in the mesial, central, and distal zones with an endodontic spreader equipped with a rubber stop. The distance from the spreader tip to the rubber stop was quantified using a digital caliper (0.01 mm accuracy) prior to de‐epithelialization and excision of the adipose tissue.
- Palatal tissue thickness was measured at the mesial, central, and distal aspects of the designated graft site using an anesthesia needle with a rubber stop. The needle was inserted perpendicular to the palatal mucosa until it contacted the underlying palatal bone. After withdrawal, the distance from the needle tip to the rubber stop was measured with a digital caliper to determine the palatal tissue thickness.

A detailed overview of the study procedures and the timing of all clinical assessments is provided in the study timeline (Figure 4).

**Figure 4:**
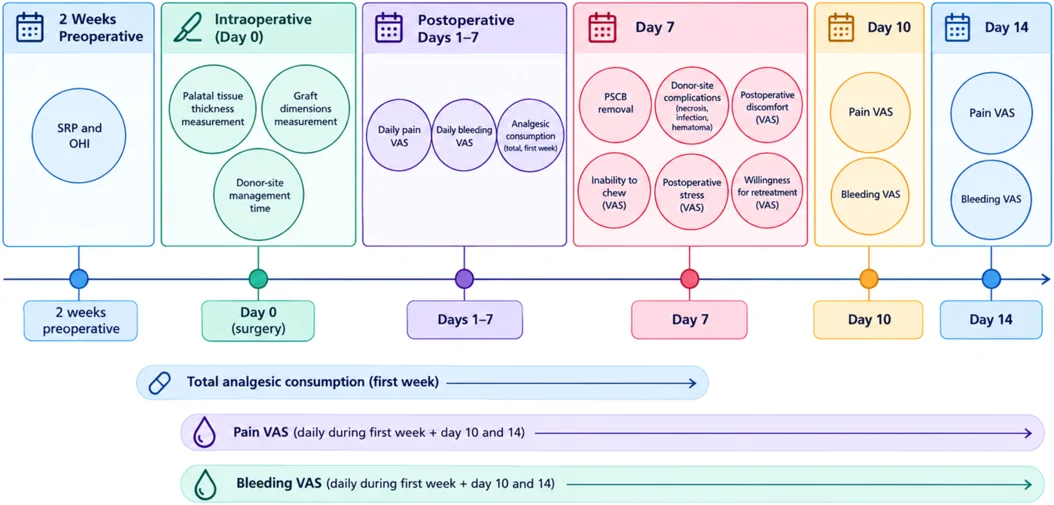
Study timeline illustrating the procedures and outcome assessments performed at each time point (OHI, oral hygiene instruction; PSCB, peripheral seal composite barrier; SRP, scaling and root planning; VAS, visual analog scale).

### 2.8 Statistical Analysis

The data were analyzed via SPSS software (SPSS version 25, IBM) by an independent statistician blinded to group allocation. Normality was checked with the Shapiro–Wilk test, and the patient served as the statistical unit. Depending on the data distribution, either the Mann–Whitney *U* test (for nonparametric variables such as daily pain, daily bleeding, discomfort, inability to chew, stress, willingness to retreat, graft width, and analgesic use) or the independent samples *t*‐test (for normally distributed variables such as palatal thickness, graft thickness, graft length, and wound management time) was applied.

In addition, a multiple linear regression analysis was performed to evaluate the effect of group allocation on postoperative pain, using total analgesic consumption as the dependent variable. Potential confounding variables, including age, sex, graft thickness, graft length, graft width, and palatal tissue thickness, were entered into the model using the enter method. A *p*‐value < 0.05 was considered statistically significant.

## 3 Results

A total of 50 patients, comprising 20 males (40%) and 30 females (60%), participated in this study. The participants were randomly allocated into control and intervention groups (*n* = 25 each). The test group included 17 females (68%) and 8 males (32%), whereas the control group included 13 females (52%) and 12 males (48%). There was no statistically significant difference in sex distribution between the groups (*p* = 0.248). The mean age was 40.92 ± 13.1 years in the test group and 41.08 ± 12.89 years in the control group, with no significant difference between the groups (*p* = 0.965). The demographic details are summarized in Table 1. All participants completed the study and attended all scheduled follow‐up visits. No dropouts were observed. The palatal soft tissue thickness and graft dimensions are presented in Table 2. No statistically significant differences in these variables were detected between the test and control groups (*p* > 0.05). Table 3 presents the mean (SD) pain scores by day and group. Pain scores were significantly lower in the intervention group than in the control group from day 1 through day 7 (*p* < 0.001). As shown in Figure 5, pain scores were significantly higher in the intervention group than in the control group on day 10 (*p* = 0.007). No statistically significant difference was observed between the groups on day 14 (*p* = 1.00). When postoperative pain was evaluated based on analgesic consumption during the first week, patients in the control group consumed significantly greater amounts of analgesics (4160.00 ± 1366.26 mg) compared to those in the test group (1328.00 ± 846.33 mg), with a *p*‐value < 0.001 (Table 4). Multiple linear regression analysis was performed to evaluate the effect of group allocation on postoperative analgesic consumption while adjusting for age, sex, graft thickness, graft length, graft width, and palatal tissue thickness. The overall model was statistically significant (*p* < 0.001; *R* ^2^ = 0.664). Group allocation remained a significant predictor of analgesic consumption (*β* = −0.788, *p* < 0.001), whereas none of the other variables showed a significant association.

**Table 1:** Demographic characteristics of the participants in the control and intervention groups.

Characteristics	Control group mean (SD) or *n* (%)	Intervention group mean (SD) or *n* (%)	*p*‐value
Age (years)	41.08 (12.89)	40.92 (13.10)	0.965
Female	13 (52.0%)	17 (68.0%)	0.248
Male	12 (48.0%)	8 (32.0%)	

**Table 2:** Graft dimensions and palatal thickness in the control and intervention groups.

Variable	Control group mean (SD) (mm)	Intervention group mean (SD) (mm)	*p*‐value
Palatal thickness	4.19 (0.31)	4.20 (0.34)	0.993^a^
Graft thickness	1.47 (0.14)	1.50 (0.13)	0.975^a^
Graft length	14.95 (2.87)	15.11 (2.80)	0.987^a^
Graft width	4.58 (0.43)	4.71 (0.41)	0.946^b^

^a^ *t*‐test independent.

^b^ Mann–Whitney *U* test.

**Table 3:** Comparison of VAS pain scores between the control and intervention groups.

Day	Control group	Intervention group	*p*‐value^a^
	Mean (SD)	Mean (SD)	
1	6.48 (1.69)	1.80 (1.26)	< 0.001
2	6.04 (1.70)	1.16 (1.14)	< 0.001
3	5.12 (1.74)	0.60 (1.04)	< 0.001
4	3.88 (1.27)	0.32 (0.75)	< 0.001
5	2.64 (1.52)	0.24 (0.72)	< 0.001
6	1.88 (1.39)	0.16 (0.55)	< 0.001
7	1.00 (1.15)	0.12 (0.44)	< 0.001
10	0.36 (0.64)	0.76 (0.52)	0.007
14	0.04 (0.20)	0.04 (0.20)	1.000

^a^ Mann–Whitney *U* test.

**Figure 5:**
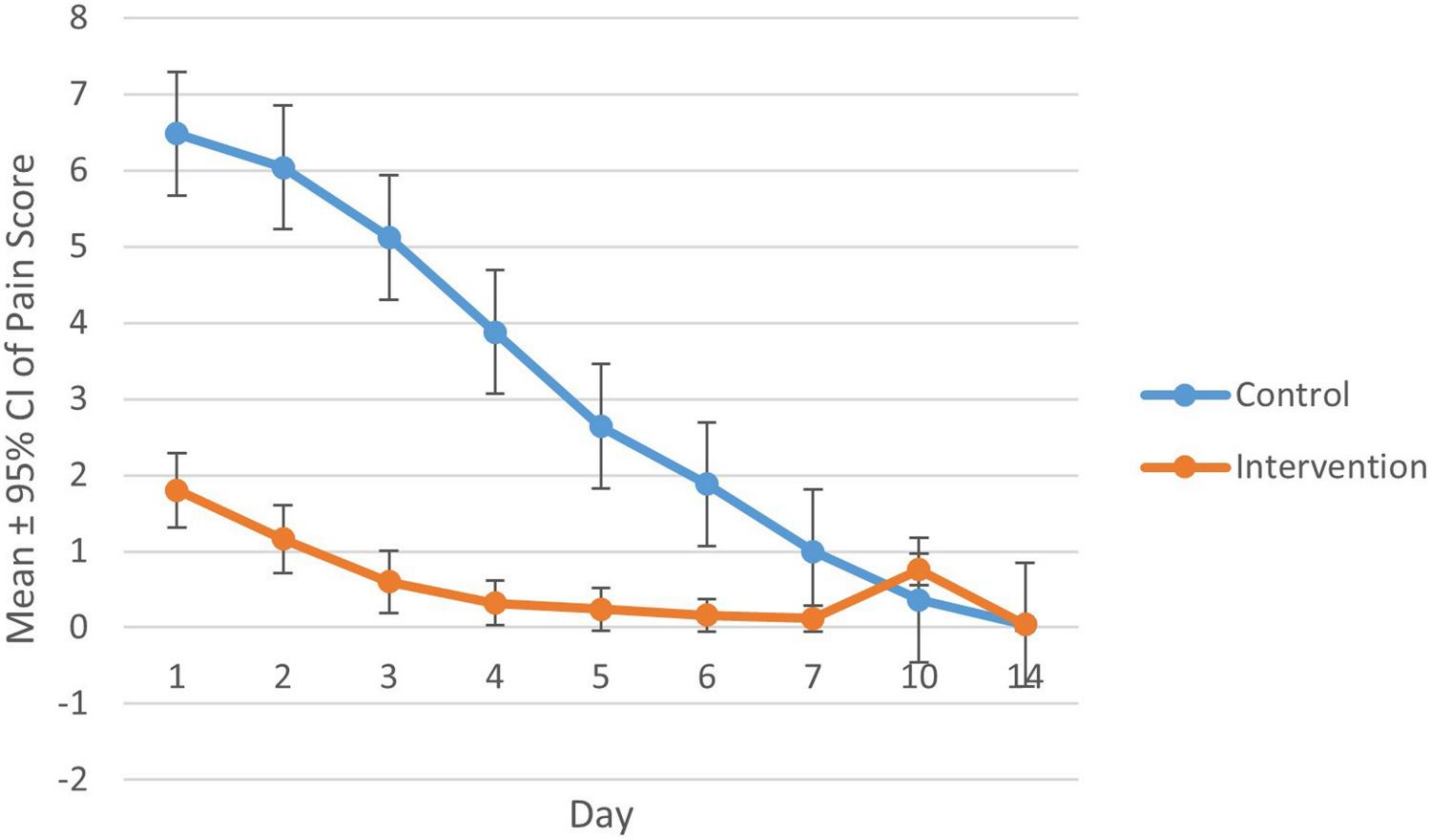
Statistical comparison of day‐to‐day pain scores between the two groups.

**Table 4:** Clinical outcomes in the control and intervention groups.

Variable	Control group mean (SD)	Intervention group mean (SD)	*p*‐value
Discomfort (VAS)	7.24 (0.93)	2.56 (1.26)	< 0.001^a^
Inability to chew (VAS)	7.24 (0.97)	3.00 (1.55)	< 0.001^a^
Stress (VAS)	7.20 (1.00)	2.04 (1.31)	< 0.001^a^
Willingness to retreat (VAS)	2.04 (1.34)	7.80 (1.15)	< 0.001^a^
Palatal management time (s)	445.76 (77.43)	210.42 (52.04)	< 0.001^b^
Analgesic consumption (mg)	4160.00 (1366.26)	1328.00 (846.33)	< 0.001^a^

^a^ Mann–Whitney *U* test.

^b^ *t*‐test independent.

As shown in Figure 6, the bleeding scores were significantly lower in the intervention group than in the control group on days 1 through 4 (*p* < 0.001). However, no significant differences in bleeding scores were observed between the groups on days 5, 6, 7, 10, and 14 (Table 5). Table 4 summarizes additional clinical outcomes. The intervention group reported significantly lower scores for postoperative discomfort (*p* < 0.001), inability to chew (*p* < 0.001), and stress (*p* < 0.001). The donor site working time was significantly shorter in the test group compared to the control group (*p* < 0.001; Table 4). In the control group, achievement of hemostasis often required repeated application of gauze pressure following initial placement of the collagen sponge and sutures. In cases of persistent bleeding, additional intervention in the form of a greater palatine compression suture was required in 8 out of 25 patients (Kulkarni et al. 2018). All such procedures were included within the recorded working time.

**Figure 6:**
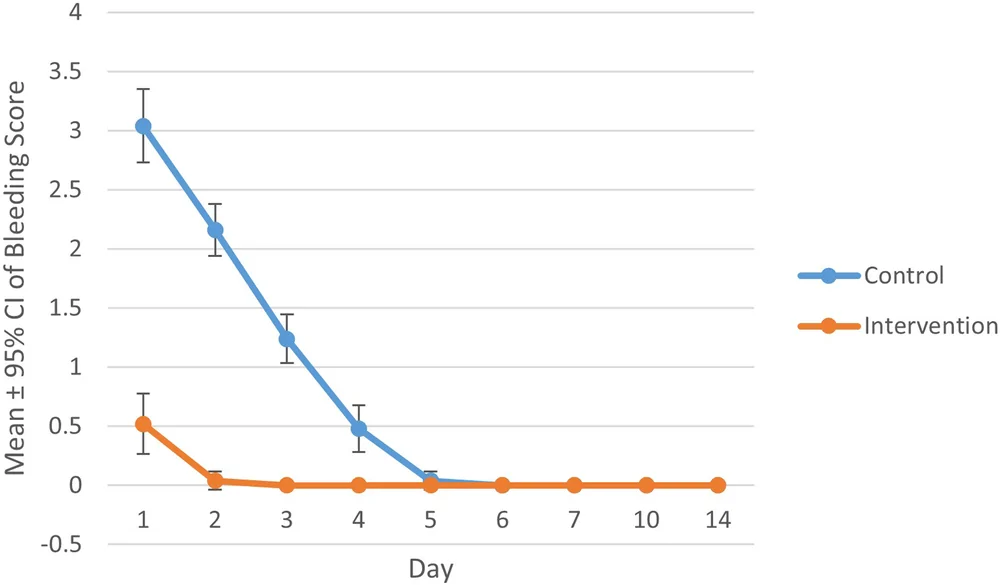
Statistical comparison of day‐to‐day bleeding scores between the two groups.

**Table 5:** Comparison of VAS bleeding scores between the control and intervention groups.

Day	Control group	Intervention group	*p*‐value^a^
	Mean (SD)	Mean (SD	
1	3.04 (0.79)	0.52 (0.65)	< 0.001
2	2.16 (0.55)	0.04 (0.20)	< 0.001
3	1.24 (0.52)	0.00 (0.00)	< 0.001
4	0.48 (0.51)	0.00 (0.00)	< 0.001
5	0.04 (0.20)	0.00 (0.00)	0.317
6	0.00 (0.00)	0.00 (0.00)	1.000
7	0.00 (0.00)	0.00 (0.00)	1.000
10	0.00 (0.00)	0.00 (0.00)	1.000
14	0.00 (0.00)	0.00 (0.00)	1.000

^a^ Mann–Whitney *U* test.

In contrast, in the test group, application of the PSCB in combination with suturing resulted in immediate stabilization of the donor site, and no additional hemostatic interventions beyond the initial procedure were required. These differences in the need for repeated and additional bleeding control measures contributed to the increased working time observed in the control group. Additionally, the willingness scores to undergo retreatment were significantly higher in the test group (7.80 ± 1.15) than in the control group (2.04 ± 1.34), as shown in Table 4 (*p* < 0.001). None of the patients in either the test group or the control group exhibited postsurgical necrosis, infection, or hematoma at the donor site. Therefore, a statistical comparison was not applicable for these variables.

## 4 Discussion

Although a variety of graft materials, such as acellular dermal matrices, collagen matrices, biologic agents, and living cellular constructs, have been employed in root coverage surgeries and soft tissue augmentations around teeth and implants, autogenous grafts, including connective tissue grafts and free gingival grafts, continue to be considered the gold standard (Tavelli, McGuire et al. 2020). The palate serves as the standard donor site for harvesting autogenous grafts, and various treatment approaches have been developed to minimize both intraoperative and postoperative morbidity associated with the donor area (Tavelli et al. 2023). This study evaluated the efficacy of using a peripheral seal composite barrier in combination with a collagen sponge and compared it to the use of a collagen sponge alone in terms of donor site management time, postoperative pain, complications, chewing ability, and delayed bleeding. Today, patient‐reported outcomes are considered a key component of successful periodontal plastic surgery (Meza‐Mauricio et al. 2023). Therefore, patients’ experiences of postoperative morbidity should be evaluated alongside clinical outcomes to provide a comprehensive assessment of treatment success (Meza‐Mauricio et al. 2021). Enhancing postoperative comfort, in addition to achieving effective clinical outcomes, has become an evolving focus in periodontal research, aiming to support overall patient well‐being (Curtis et al. 1985). In line with this, the present study incorporated patient‐reported outcome measures (PROMs) to evaluate the subjective experiences of patients following surgery.

In this study, our findings demonstrated that using PSCB (in addition to a collagen sponge) as a mechanical protection for palatal wounds significantly reduced postoperative complications and improved patient satisfaction following graft harvesting compared with the use of a hemostatic sponge alone. This result is in agreement with a previous study reporting that covering hemostatic collagen sponges with flowable resin composites significantly and clinically reduced postoperative pain (Meza‐Mauricio et al. 2023). Some factors related to patients and surgical procedures can potentially affect postoperative pain and discomfort at the palatal donor site, including smoking, palatal soft tissue thickness, and the thickness of the harvested graft (Tavelli et al. 2023). According to Burkhardt et al. (2015) palatal thickness less than 4 mm and graft thickness greater than 2 mm are associated with greater postoperative pain following FGG harvesting. To eliminate potential confounding effects, smokers were excluded from participation in our study. In this study, the graft thicknesses were 1.50 ± 0.13 mm in the test group and 1.47 ± 0.14 mm in the control group, which were less than 2 mm in both groups, also, there were no significant differences between the groups. Additionally, the average palatal soft tissue thickness in both groups was greater than 4 mm. The palatal soft tissue thickness was 4.20 ± 0.34 mm in the test group and 4.19 ± 0.31 mm in the control group, with no statistically significant difference between the groups. To our knowledge, no studies have investigated whether pain at the recipient site may influence pain perception in the palatal donor area. However, to minimize potential bias in identifying the source of pain (recipient or donor site), grafts were harvested from the side contralateral to the recipient site (Meza‐Mauricio et al. 2023). This study demonstrated that patients treated with PSCB experienced significantly less pain, as measured by VAS scores, and required fewer analgesics during the first postoperative week (*p* < 0.001), which is typically the most painful period following surgery (Burkhardt et al. 2015). This may be attributable to the protective effect of PSCB, which minimizes irritation and trauma to the wound during the early healing phase. However, following removal of the composite shield, the pain scores in the test group were greater than those in the control group on postoperative day 10 (*p* = 0.007). The observed increase in pain at day 10 in the test group may be related to the timing of PSCB removal at day 7, which coincides with a sensitive phase of wound healing and increased exposure of the donor site. Following removal of the protective barrier, the palatal tissue may be more susceptible to mechanical irritation during mastication and oral hygiene procedures, potentially resulting in transient discomfort. In addition, a relative rebound in pain perception may occur after an initial period of reduced symptoms in the early postoperative phase. While a delayed healing response associated with the presence of the composite barrier cannot be excluded, this finding should be interpreted with caution, and further studies are needed to better understand the effect of composite materials on palatal wound healing.

When evaluating intraoperative and postoperative bleeding, it is important to consider that severe and excessive bleeding is often the result of direct injury to the greater palatine artery (Tavelli et al. 2023). Management of this complication typically involves applying firm pressure on the area for several minutes, placing a deep compressive suture distal to the donor site (Kulkarni et al. 2018), or performing electrocauterization. A thorough understanding of palatal anatomy and identification of the greater palatal foramen by palpation are essential to help prevent such vascular injuries (Tavelli, Barootchi et al. 2019; Shahbazi et al. 2019). However, excessive bleeding during surgery is not always related to direct greater palatine artery injury (Tavelli et al. 2023). A previous study demonstrated that the graft harvesting technique itself plays a critical role in the amount of intraoperative bleeding (Tavelli, Barootchi et al. 2020). This study demonstrated that harvesting subepithelial CTGs via the trapdoor technique was associated with more fluid leakage and more vessel injury than DGGs (Tavelli, Barootchi et al. 2020). To achieve hemostasis and bleeding control after graft harvesting, various hemostatic agents, such as absorbable gelatin sponges, oxidized regenerated cellulose, and ferric subsulfate, have been used in previous studies (Rossmann and Rees 1999; Burkhardt et al. 2015). Additionally, studies have shown that using a mechanical barrier such as propylene mesh or applying cyanoacrylate to the donor site significantly reduces the time required to achieve hemostasis (Yussif et al. 2021; Ozcan et al. 2017). Similarly, in the present study, the time required to achieve hemostasis was significantly shorter in the test group than in the control group (*p* < 0.001). This outcome may be attributed to the peripheral seal created by the PSCB, which closely adapts to the surrounding palatal tissues and enhances hemostasis by exerting localized compressive forces. This close adaptation helps prevent blood leakage and promotes the rapid formation of a stable clot at the donor site. Additionally, patients in the test group experienced significantly less delayed bleeding during the first four postoperative days than did those in the control group (*p* < 0.001). The PSCB provided both rapid and sustained hemostasis.

Collagen sponges play a pivotal role in the management of palatal donor sites. They offer several favorable characteristics, including inherent biocompatibility, low antigenicity, ease of handling, and cost‐effectiveness (Chattopadhyay and Raines 2014). They are widely used in the management of palatal donor sites, primarily for achieving hemostasis and supporting wound healing. However, to achieve complete sealing and isolation of the wound from the oral environment, covering the collagen sponge with additional materials has been proposed (Tavelli et al. 2018; Laguna‐Martos et al. 2025). Examples include the use of flowable composite resin or cyanoacrylate, both of which have been shown in previous studies to be effective in managing and reducing postoperative pain at the donor site (Meza‐Mauricio et al. 2023; Tavelli, Ravidà, Saleh et al. 2019). Additionally, in this study, flowable composite resin was used to cover the collagen sponge. However, the proposed method differs slightly from previously reported techniques. In the study by Meza‐Mauricio et al. (2023), the flowable composite was injected directly onto the collagen sponge that was placed on the palatal wound and then light‐cured. The approach used in the present study involves fabricating the composite barrier before graft harvesting and prior to any incision. Therefore, the composite barrier is constructed over intact palatal tissue and extends approximately 2–3 mm beyond the anticipated graft area. This preoperative fabrication prevents direct contact between uncured composite monomers and the open wound, thereby minimizing the risk of tissue irritation or damage. In addition, extending the PSCB beyond the wound margins contributes to more effective sealing of the donor site, facilitates faster intraoperative bleeding control, promotes stable clot formation, and reduces the risk of delayed postoperative bleeding. Unlike previous methods, this technique does not require bonding the PSCB to the palatal surfaces of adjacent teeth (Gisotti and Valente 2024; Laguna‐Martos et al. 2025). Instead, the composite barrier is secured solely with a sling cross mattress suture. This eliminates the need for enamel etching and bonding procedures or composite removal, simplifying both the placement and removal process of PSCB, reducing surgical time, and improving patient comfort.

The findings of this study are consistent with previous studies demonstrating that covering collagen sponges with materials such as composite or bioadhesive materials can effectively reduce postoperative pain and discomfort (Meza‐Mauricio et al. 2023; Tavelli, Ravidà, Saleh et al. 2019). This benefit is likely attributable to the physical barrier created by the composite, which helps isolate the wound from mechanical and chemical irritants present in the oral environment. Similarly, a study by Basma et al. (2023) demonstrated that the use of a palatal stent was associated with reduced overall pain, lower consumption of pain killers, and greater willingness among patients to undergo the same procedure again. Also, another study showed that complaints related to the donor site after graft harvesting may decrease with the use of coverage techniques, including Essix or Hawley retainers (Eltas et al. 2014). Additionally, in the present study, patients in the test group reported significantly greater willingness to undergo a similar treatment if needed (*p* < 0.001). Additionally, the reported VAS scores for postoperative discomfort, stress, and chewing difficulty were significantly lower in the test group compared to the control group (*p* < 0.001). None of the patients in this study experienced postoperative complications such as necrosis, infection, or hematoma at the palatal donor site during the overall postoperative period.

One notable advantage of PSCB over other mechanical protection methods, such as Essix retainers, stents, or Hawley appliances, is its ease of chairside fabrication. In contrast, these traditional approaches typically require laboratory fabrication, which is time‐consuming and costly (Gisotti and Valente 2024). Additionally, these appliances are often bulky, covering multiple teeth and large areas of the palate, which may interfere with patient comfort and function (Wyrebek et al. 2018). However, the PSCB is made from a relatively thin layer of flowable composite resin, does not interfere with occlusion, does not cover adjacent teeth, and is designed to extend only 2–3 mm beyond the wound margins. This allows for localized coverage of the donor site, enhancing patient comfort without unnecessary bulk. Notably, in this study, none of the patients reported any complaints regarding the size or bulkiness of the composite material. Moreover, the composite resin used to fabricate the PSCB has a smoother surface texture compared to the acrylic material traditionally used in conventional appliances. This feature limits plaque accumulation and tissue irritation, enhancing patient comfort. A potential limitation of the PSCB technique is the risk of fracture or dislodgement of the composite barrier, as it is applied in a relatively thin layer. Although no such complication was observed in any of the patients included in this study, it is recommended that patients adhere to a soft diet and avoid applying pressure to the area during the early healing phase. Another important consideration is that the PSCB is fabricated prior to the graft harvesting procedure. Therefore, if any intraoperative adjustments in the size or shape of the graft become necessary, the preformed PSCB may no longer perfectly match the wound dimensions. This mismatch could compromise the adaptation and sealing capacity of the barrier.

One of the limitations of this study is that wound healing status was not systematically assessed or compared between the two groups. the use of standardized indices, such as the Landry Wound Healing Index (Landry 1985) or the hydrogen peroxide test (Marucha et al. 1998), could provide a more comprehensive evaluation of healing outcomes and the potential effects of composite barrier. Therefore, future randomized controlled trials are needed to evaluate this aspect more thoroughly. Another limitation of this study is that individual pain thresholds were not assessed prior to enrollment of the patients in the study (Gatti et al. 2023). Pain perception varies significantly among individuals, which may influence postoperative pain reported by tools such as the VAS. Additionally, some patients may be reluctant to take analgesics, so indirect pain assessment based on analgesic consumption may not always provide a fully reliable measure of the actual pain experience (Gatti et al. 2023). In this study, we also assessed postoperative stress (which could also impact pain perception), discomfort, and chewing ability. These additional patient‐reported outcomes enhance the comprehensiveness of the postoperative evaluation.

## 5 Conclusion

The use of the peripheral seal composite barrier, as a modification of previously introduced chairside composite barriers for protecting the palatal donor site, significantly reduces the time required for palatal wound management and bleeding control. The PSCB technique significantly reduces patient‐reported pain and discomfort and enhances the overall patient experience of periodontal plastic surgeries. PSCB improves patients’ chewing ability and their willingness to undergo similar procedures in the future if needed. Its easy chairside fabrication, minimal bulk, and patient tolerability make it a practical clinical option. By providing a close peripheral seal, the PSCB helps prevent blood leakage, accelerates the formation of a stable clot, and supports hemostasis during the early healing phase. However, further studies are needed to assess the potential effects of the composite barrier on epithelialization and the healing process.

## Author Contributions

Moein Khojaste contributed in conceptualization, methodology, investigation, data curation, and writing. Farid Shiezadeh contributed in methodology, validation, supervision, and project administration. Zahra Moslehitabar contributed in conceptualization, investigation, and writing – editing the main manuscript. All the authors reviewed the final draft of the manuscript.

## Funding

The authors have nothing to report.

## Ethics Statement

The study protocol was approved by the Research and Ethics Committee of Mashhad University of Medical Sciences (IR.MUMS.DENTISTRY.REC.1404.032). The study was conducted in accordance with all relevant principles of the Declaration of Helsinki as revised in 2013.

## Consent

All patients provided written informed consent before enrollment.

## Conflicts of Interest

The authors declare no conflicts of interest.

## Data Availability

The data of this study can be presented upon reasonable request.
